# Comparative proteomic analysis of gingival crevicular fluid and periodontal tissue: revealing clinical potential

**DOI:** 10.1186/s12014-026-09587-3

**Published:** 2026-02-24

**Authors:** Jeong-hun Mok, Ji-Youn Hong, MinJoong Joo, Won Seok Bang, Do-Young Ahn, Jeong-Ho Yun, Jong-Moon Park

**Affiliations:** 1 Department of Medical Device Management and Research, SAIHST, Sungkyunkwan University, Seoul, 06355 Republic of Korea; 2 College of Pharmacy, Gachon University, Incheon, 21936 Republic of Korea; 3 Department of Periodontology, College of Dentistry, Kyung Hee University Dental Hospital, Kyung Hee University, Seoul, 02447 Republic of Korea; 4 Basilbiotech, Incheon, 22002 Republic of Korea; 5 Department of Periodontology, College of Dentistry, Institute of Oral Bioscience, Jeonbuk National University, Jeonju, 54907 Republic of Korea; 6 Research Institute of Clinical Medicine of Jeonbuk National University-Biomedical Research Institute of Jeonbuk National University Hospital, Jeonju, 54907 Republic of Korea

**Keywords:** Periodontitis, Proteomic profiling, GCF, Neutrophil, Biomarker discovery

## Abstract

**Background:**

Periodontitis is a chronic inflammatory disease characterized by tissue destruction and immune dysregulation. While gingival crevicular fluid (GCF) serves as a non-invasive biomarker source, its molecular distinctions from periodontal tissue remain underexplored. This study conducted a comparative proteomic analysis of GCF and tissue samples from patients with Stage III–IV periodontitis, integrating differential expression, weighted gene co-expression network analysis, and protein–protein interaction networks to delineate compartment-specific molecular profiles and clarify their respective biological roles in periodontal pathophysiology.

**Methods:**

Proteomic data were acquired from GCF and periodontal tissue using label-free LC–MS analysis. Differentially expressed proteins (DEPs) were identified using independent samples t-test (*p* < 0.05, |fold-change| > 2). WGCNA was performed to construct co-expression modules and identify functionally related protein clusters, followed by GO enrichment and protein–protein interaction (PPI) analyses using STRING and Cytoscape. Hub proteins were determined through CytoHubba according to centrality measures. Comparative analyses were conducted between tissue and GCF to define inflammation- and repair-related modules and to assess potential molecular interconnections between the two sample types.

**Results:**

A total of 4,^1^04 proteins were identified in periodontal tissue and 1,546 in GCF. Among these, 1,292 DEPs were detected in tissue and 280 in GCF. Periodontal tissue displayed coordinated upregulation of ribosomal proteins and collagen networks alongside mitochondrial components, indicating repair-oriented structural remodeling and metabolic activation. Conversely, GCF exhibited enrichment of neutrophil-derived immune effectors including MPO, ELANE, CTSG, S100A8, and apolipoproteins, representing innate immune activation. Network integration revealed that GCF and tissue maintained largely distinct molecular profiles with limited cross-compartment connectivity.

**Conclusions:**

This comparative proteomic analysis demonstrates that periodontal tissue and GCF represent functionally distinct but complementary biological environments in periodontitis. Periodontal tissue exhibits enhanced structural and metabolic processes, whereas GCF predominantly reflects neutrophil-mediated immune responses. These molecular distinctions provide a basis for developing clinical, compartment-specific biomarker candidates that warrant analytical validation, thereby supporting precision-medicine-relevant diagnostic strategies in periodontal research.

**Supplementary Information:**

The online version contains supplementary material available at 10.1186/s12014-026-09587-3.

## Background

Periodontitis is a common chronic inflammatory disease that progressively damages the gingiva, periodontal ligament, and alveolar bone, ultimately leading to tooth loss if untreated [1, 2]. The early stages are typically asymptomatic, allowing subclinical inflammation and tissue degradation to accumulate undetected. This highlights the need for analytical approaches capable of revealing early molecular changes in the periodontium [3, 4].

Among various biological fluids, gingival crevicular fluid (GCF)^1^ offers a unique opportunity for non-invasive molecular exploration, as it originates at the interface between the gingival epithelium and the tooth surface and reflects the local inflammatory milieu [5–7]. Unlike periodontal tissue, GCF contains immune-related proteins regulated by external factors, such as microbes, making it a valuable source for identifying clinically relevant biomarkers for periodontitis [8, 9]. Proteomic profiling of GCF has emerged as a promising strategy to uncover immune-related proteins dynamically regulated in response to microbial and host-derived signals, offering insight into periodontal pathophysiology and host-microbiome interactions without relying on invasive tissue sampling [10, 11]. While studies have analyzed GCF or periodontal tissue separately, comparative analyses remain limited, warranting investigation of their distinct molecular contributions [12–15]. For instance, matrix metalloproteinases (MMPs) are among the most frequently suggested proteins in periodontal biomarker research and are often regarded as molecular indicators of periodontal tissue degradation [16]. However, such interpretations frequently lack direct evidence verifying the precise tissue source of these enzymes. GCF is a complex biological fluid that contains both tissue-derived proteins and immune cell secretions, particularly those released by neutrophils, including MMP-8 and MMP-9 [17]. Although previous studies have analyzed GCF or periodontal tissue independently, the distinct molecular contributions of each sample type to periodontal pathophysiology remain unclear.

GCF constitutes a complex exudate comprising serum-, tissue-, and biofilm-derived components [18, 19], with measurable flow and constitutive neutrophil trafficking reported even at clinically healthy sites [20] despite opposing observations [21]. Previous histological studies have demonstrated basement-membrane-limited passage toward the sulcus [22, 23], consistent with epithelial transport and osmotic models [24] and with inflammation-induced increases in vascular permeability and Starling forces that enhance plasma filtration and interstitial pressure [25–27]. Given this inherent compositional overlap and cross-compartment exchange, source attribution of detected proteins cannot be assumed, necessitating a compartment-specific comparative proteomic analysis to delineate source-specific molecular signatures.

In this study, we conducted a comparative proteomic analysis of GCF and periodontal tissue samples from patients with periodontitis, with the aim of delineating the distinct molecular contributions of each sample type to disease progression. Through differential expression profiling, Weighted Gene Co-expression Network Analysis (WGCNA), and protein-protein interaction (PPI) mapping, we identified functionally relevant protein modules associated with inflammatory activity and structural remodeling. This study provides molecular insights into the distinct roles of GCF and tissue in inflammation and repair, thereby supporting the identification of biomarker candidates and informing diagnostic strategies that address both immune activation and structural remodeling in periodontitis.

## Materials and methods

### Materials

HPLC grade water, acetonitrile, methanol, and isopropanol were obtained from JT Baker (Philipsburg, NJ, USA). Urea, iodoacetamide (IAA), formic acid (FA), and ammonium bicarbonate (ABC) were purchased from Sigma-Aldrich (St. Louis, MO, USA). Tris(2-carboxyethyl) phosphine (TCEP) was purchased from Thermo Fisher Scientific (Rockford, IL, USA). Sequencing-grade modified trypsin was purchased from Promega (Madison, WI, USA).

## Sample Preparation

This study recruited patients from our university hospital who provided informed consent for the use of discarded tissues. The study protocol was approved by the Ethics Committee of the Jeonbuk National University Hospital (approval no. CUH 2015-06-038) and Kyung Hee University Dental Hospital (IRB no. KH-DT23015). The study protocol conforms to the guidelines of Good Clinical Practice and the Declaration of Helsinki. All participants provided written informed consent. Tissue samples in the healthy control group were obtained from teeth extracted for orthodontics treatment or third molar removal, without clinical signs of inflammation. For periodontal tissue sampling, the periodontitis group comprised patients requiring tooth extraction owing to advanced periodontal disease, characterized by bleeding on probing (BOP), a probing depth (PD) of greater than or equal to 5 mm, and radiographic evidence of severe alveolar bone loss (≥ 2/3 of supporting structure). Exclusion criteria for both periodontitis and control group encompassed the following: heavy smoking (≥ 10 cigarettes/day), use of bone metabolism-altering medications, current rheumatoid arthritis therapy, bisphosphonate treatment exceeding 3 months prior to extraction, uncontrolled diabetes or hypertension, history of head and neck radiation, pregnancy or lactation, and substance dependence. Following the extraction, the periodontal ligament and connective tissue adherent to the tooth root were excised and scraped with a #15 surgical blade under same conditions for both the periodontitis and control group. In addition, tissue volume was adjusted to match those of the control group to account for the additional inflamed tissue present in the periodontitis group. Inflamed tissue was first excised in its entirety and then combined with the adherent to the periodontal ligament of the tooth root to achieve a total tissue volume comparable to that of the control group. The periodontal tissue samples were immersed in phosphate-buffered saline for storage (Sigma-Aldrich) and heat-stabilized using the Stabilizer T1 system (Denator AB, Gothenburg, Sweden). Frozen periodontal tissue samples were cryopreserved using a CP02 Automated Dry Pulverizer (Covaris, Woburn, MA, USA).

Periodontal GCF samples were obtained from patients diagnosed with Stage III or IV periodontitis according to the 2017 World Workshop on the Classification of Periodontal Diseases [28]. For each patient, the tooth with the deepest PD at interproximal sites was selected, exhibiting a mean PD of 7.63 ± 0.92 mm and clinical attachment loss of 8.00 ± 0.76 mm. All the patients exhibited 100% BOP, indicating active periodontal inflammation. The GCF was collected using absorbent paper points (Meta IBiomed, Chungju, Korea) and inserted into the sulcus for 30 s. Contaminated paper points were discarded to ensure uncontaminated samples. GCF samples from the healthy control group were defined as those obtained from subjects with PD < 4 mm and BOP ≤ 10% across all tooth sites.

Both pulverized periodontal tissue samples and GCF were sonicated for protein extraction using a Covaris S2 Focused-Ultrasonicator (Covaris) in lysis buffer (8 M urea, 0.1 M Tris-HCl, pH 8.5). Protein concentrations were measured using a bicinchoninic acid Protein Assay Kit (Pierce Biotechnology, Rockford), and 100 µg of protein was used for digestion. Protein digestion was performed following the filter-aided sample preparation technique using a Microcon 30 K centrifugal filter unit (Millipore, Billerica, MA, USA). Proteins were reduced with 5 mM TCEP at 37 °C for 30 min, alkylated with 50 mM IAA at 25 °C for 1 h in the dark, washed with lysis buffer and 50 mM ABC, and digested with trypsin (enzyme-to-protein ratio of 1:50, w/w) at 37 °C for 18 h. The resulting peptides were transferred to new tubes, the pH was adjusted to 2–3, desalted using a C18 spin column (Harvard Apparatus, Holliston, MA, USA), and dried using a centrifugal concentrator (SCANVAC; LaboGene Aps, Lynge, Denmark). Dried residues were reconstituted in 100 µL of 0.1% FA, mixed, centrifuged to remove debris, and stored at − 20 °C until LC-MS analysis.

## LC-MS analysis

Proteomic analyses were carried out using a nano-LC-MS/MS using Q Exactive™ Hybrid Quadrupole-Orbitrap MS equipped with a Dionex Ultimate 3000 HPLC system (Thermo Fisher Scientific, San Jose, CA, USA). To LC-MS analysis, the injection volume was set at 1 µg. Each peptide digests were loaded onto an Acclaim™ Pep-Map™ 100 C18 nano-trap column (75 μm × 2 cm, 3 μm particles, 100 Å pore size) and eluted using a mobile phase at a flow rate of 3 µL/min for 10 min. Following the trapping phase, separation was conducted on an Acclaim™ PepMap™ C18 RSLC nano-column (75 μm × 50 cm, 2 μm particles, 100 Å pores, Thermo Fisher Scientific, San Jose) using a mobile phase consisting of 0.1% FA in water (solvent A) and 0.1% FA in 80% acetonitrile (solvent B) at a constant flow rate of 0.3 µL/min. The solvent gradient was configured as follows: start at 4% solvent B for 14 min, increase to 20% solvent B over 61 min, then to 50% solvent B over 70 min, rapidly increase to 96% solvent B for 2 min, maintain 96% solvent B for 13 min, drop back to 4% solvent B over 1 min, and hold at 4% solvent B for 24 min. ESI parameters were set with a spray voltage of 2.2 kV and a capillary temperature of 320 °C.

### Data analysis

Thermo MS raw files were analyzed using Proteome Discoverer 2.5.0.400 software (Thermo Fisher Scientific, San Jose). The search parameters included a maximum of two missed cleavages with trypsin, 10 ppm and 0.02 Da tolerances of precursor ion masses and fragment ion mass, respectively, carbamidomethylation of cysteine (+ 57.012 Da), methionine oxidation (+ 15.995 Da), and carbamylation of protein in the N-terminus (+ 43.0006 Da). Peptide identifications were filtered at 1% FDR using the Target Decoy PSM Validator module in Proteome Discoverer. Protein identifications were filtered to include those with at least two unique peptides and 1% protein-level FDR. The *Homo sapiens* database was obtained from UniProt (released 02, 2015). Protein concentration data based on intensities for related quantitative analyses was normalized to the total sum of all protein intensities after imputation to remove missing values, followed by a log10 transformation.

### Statistical analysis

**Fig. 1:**
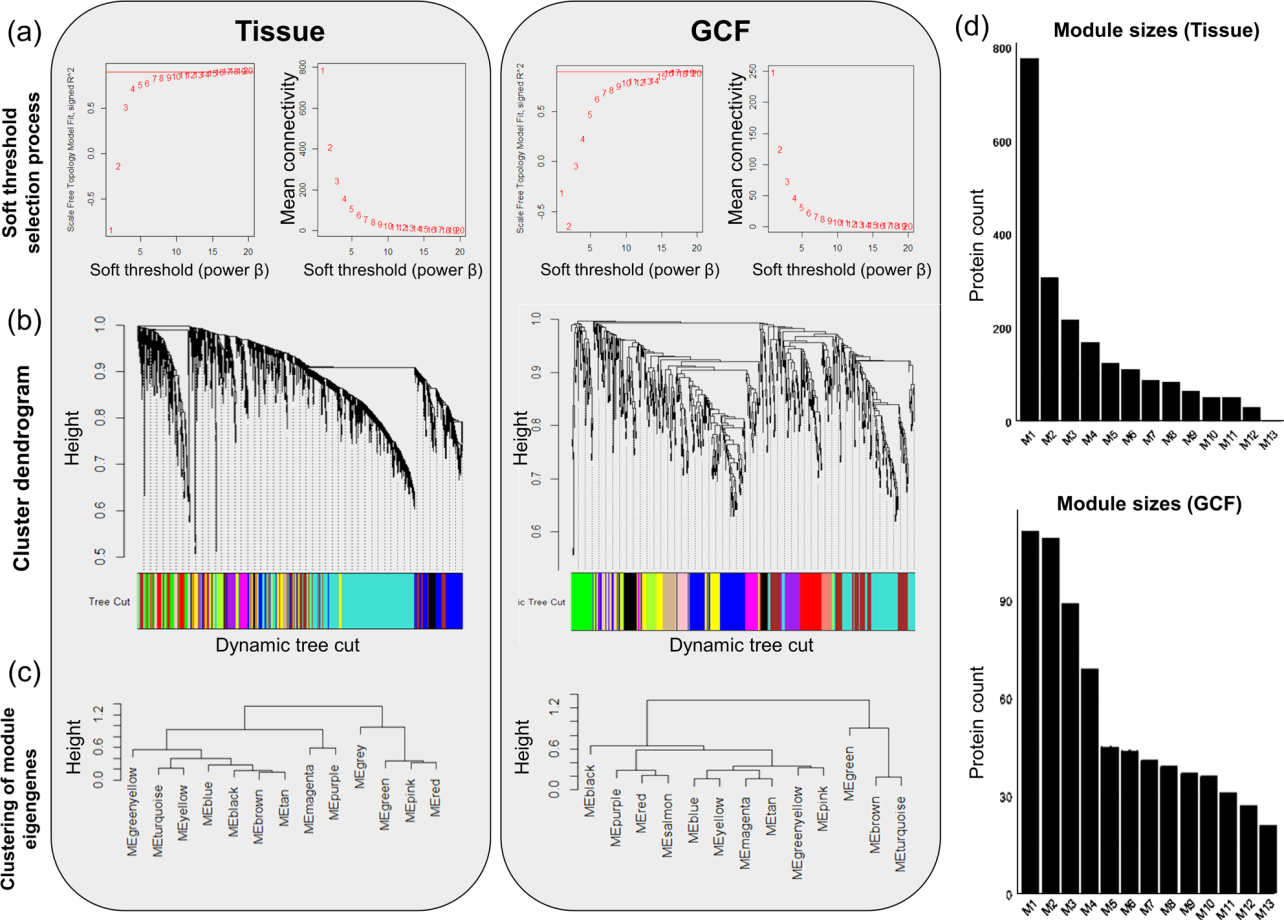
Weighted Gene Co-expression Network Analysis of periodontal tissue (*n* = 18) vs. GCF proteomes (*n* = 7). (**a**) Soft-thresholding power (β) using the pickSoftThreshold function to achieve a scale-free topology. A power of 16 was selected, indicated by the red line, with a scale-free topology fit index (R²) exceeding 0.9 and mean connectivity values stabilizing. (**b**) Construction of the Topological Overlap Matrix and subsequent cluster dendrogram based on differentially expressed proteins in periodontitis tissue and GCF samples. Branches of the dendrogram represent different co-expression modules identified using dynamic tree cut (deepSplit = 2, minClusterSize = 20). Modules are represented by distinct colors beneath the dendrogram. (**c**) Module eigengene clustering dendrogram based on similarity, visualizing module relationships and hierarchical clustering, indicating co-expression patterns. (**d**) Bar graph visualization of module sizes, ordered by the number of proteins in each module (M1–M13). The y-axis represents the number of protein count

The differentially expressed proteins (DEPs) in each group were selected for WGCNA using the R package “WGCNA” [29, 30]. The soft-thresholding power (β) was determined using the ‘pickSoftThreshold’ function, optimizing the scale-free topology fit index (R²) and mean connectivity (Fig. 1a). A power of 16 was selected to achieve an R² greater than 0.9. A topological overlap matrix was constructed, followed by a cluster dendrogram (Fig. 1b). Modules were defined using the dynamic tree-cut algorithm (deepSplit = 2, minClusterSize = 20) and visualized using distinct colors. Module eigengenes were clustered by height to evaluate similarity (Fig. 1c), and modules were ordered by size (M1–M13) and visualized using a bar graph (Fig. 1d). Then, GO enrichment analysis of the top five modules focused on biological processes using DAVID bioinformatics resources. PPI networks were visualized using Cytoscape v3.9.1 (https://cytoscape.org/), with disconnected nodes hidden and a confidence score greater than 0.4. MCODE clustering was run using default parameters, and STRING-based GO enrichment (version 11.5) provided functional annotations. Hub proteins were identified in each PPI network using CytoHubba and ranked according to the centrality measures (MCC, DMNC, degree, closeness, and betweenness).

## Results

### Differential expression patterns in GCF and tissue

In this study, we profiled the proteomes of periodontal tissues (*n* = 23; periodontal tissue = 18, control = 5) and GCF samples (*n* = 13; periodontitis GCF = 7, control = 6) collected from patients with periodontitis. A total of 4,104 proteins were identified in periodontal tissue samples and 1,546 proteins were identified in GCF samples. DEPs were identified using an independent samples *t*-test (*p* < 0.05) and a fold-change (FC) threshold of |FC| > 2 (Supplementary Table S1 and S2). In the tissue group, 1,292 DEPs were identified (1,134 upregulated and 158 downregulated), and 280 DEPs were identified in the GCF group (123 upregulated and 157 downregulated). Subsequently, we performed WGCNA on the DEPs of periodontitis tissues and GCF to identify biological changes between patients with periodontitis and controls. The analysis identified 13 modules with co-expression traits in both tissues and GCF (Supplementary Table S3 and S4).

**Fig. 2:**
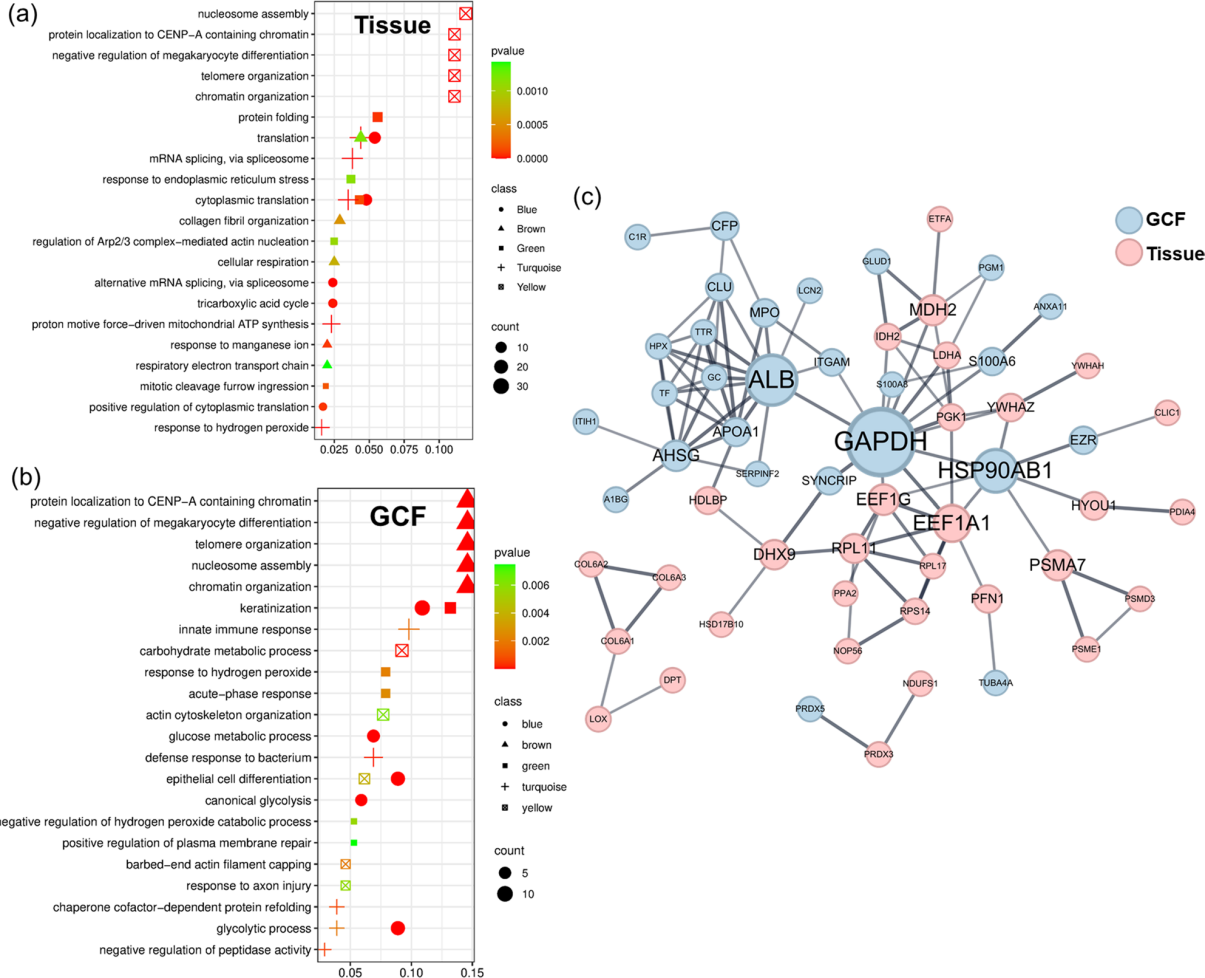
(**a**,** b**) Dot plots of GO enrichment for WGCNA modules in the tissue (**a**) and GCF (**b**) groups. Shapes represent modules: Circle (Blue), triangle (Brown), square (Green), cross (Turquoise), X-marked square (Yellow). X-axis: GeneRatio; Y-axis: GO terms description. Symbol size reflects gene count; color scale indicates –log10(p-value), from green (low) to red (high) significance. (**c**) Integrated protein-protein interaction network of hub proteins based on WGCNA from GCF and periodontal tissue. Network analysis using STRING database showing hub proteins from GCF (light blue nodes) and periodontal tissue (light red nodes). Node sizes are proportional to betweenness centrality values

The key findings in periodontal tissue samples revealed an upregulation of protein synthesis, accompanied by alterations in energy metabolism and cellular structural components, compared to healthy controls (Fig. 2a). The cytoplasmic translation and translation pathways were the most notable in both the M1 and M2 modules, indicating active protein synthesis in the tissue. Key hub proteins in the translation-related modules included ribosomal proteins (RPL11, RPL17, RPS14) and translation elongation factors (EEF1A1, EEF1G), along with glycolytic enzymes (PGK1, LDHA) and proteasomal components (PSME1, PSMA7), listed in Supplementary Table S5. The identification of mitochondrial and mRNA splicing-related pathways further highlighted the importance of energy metabolism and mRNA processing in protein synthesis, with hub proteins including NDUFS1 (NADH dehydrogenase), mitochondrial enzymes (IDH2, MDH2), and antioxidant proteins (PRDX3). Additionally, collagen fibril organization in M3 was characterized by extracellular matrix proteins including COL6A1, COL6A2, COL6A3, and proteoglycans (ASPN, DPT, OGN), while chromatin organization and nucleosome assembly in M5 emphasized considerable changes in the cell structure. The ER-related module featured proteins involved in protein folding and trafficking (PDIA4, SEC24A, LMAN2).

Immune response processes were predominant in GCFs (Fig. 2b). M1 features a defense response to bacteria and an innate immune response, whereas M5 highlighted an acute-phase response, indicating active bacterial defense and innate immunity. Glycolytic processes in M2 and carbohydrate metabolic processes in M4 were the key processes identified in the GCF. Taken together, the GCF samples exhibited strong immunological traits, with upregulation of bacterial defense mechanisms and innate immune responses, along with alterations in carbohydrate metabolism. WGCNA analysis further identified module-specific hub proteins based on kME values, with S100A8, LCN2, ALB, and APOA1 emerging as key hub proteins in defense-related modules (M1, M5), while EEF1A1, HSP90AB1, PGM1, and TTR were identified as hub proteins in metabolic modules (M2, M4) (Supplementary Table S5). GO enrichment analysis confirmed distinct functional profiles between GCF and periodontal tissue samples, corroborating the proteomic differences observed in the initial comparative analysis.

Additionally, considering that the two sample types cannot be completely isolated compartments in periodontal pathophysiology, we further investigated how GCF might reflect tissue status by exploring potential connecting mechanisms between them. Hub proteins from each group were integrated into a unified PPI network using the STRING database, and proteins with high betweenness centrality were identified to explore potential cross-interaction points (Supplementary Table S6, Fig. 2c). Network analysis revealed that interactions between GCF and tissue hub proteins were predominantly mediated by a limited number of proteins with notably high betweenness centrality scores. In the GCF group, GAPDH showed the highest betweenness centrality (1450), followed by ALB (883), HSP90AB1 (634), and APOA1 (120). From the periodontal tissue group, EEF1A1 (412) and EEF1G (194) demonstrated the most prominent bridging characteristics, with only these select proteins showing substantial connectivity between the two sample types.

### Comparison between periodontal tissue and GCF

**Fig. 3:**
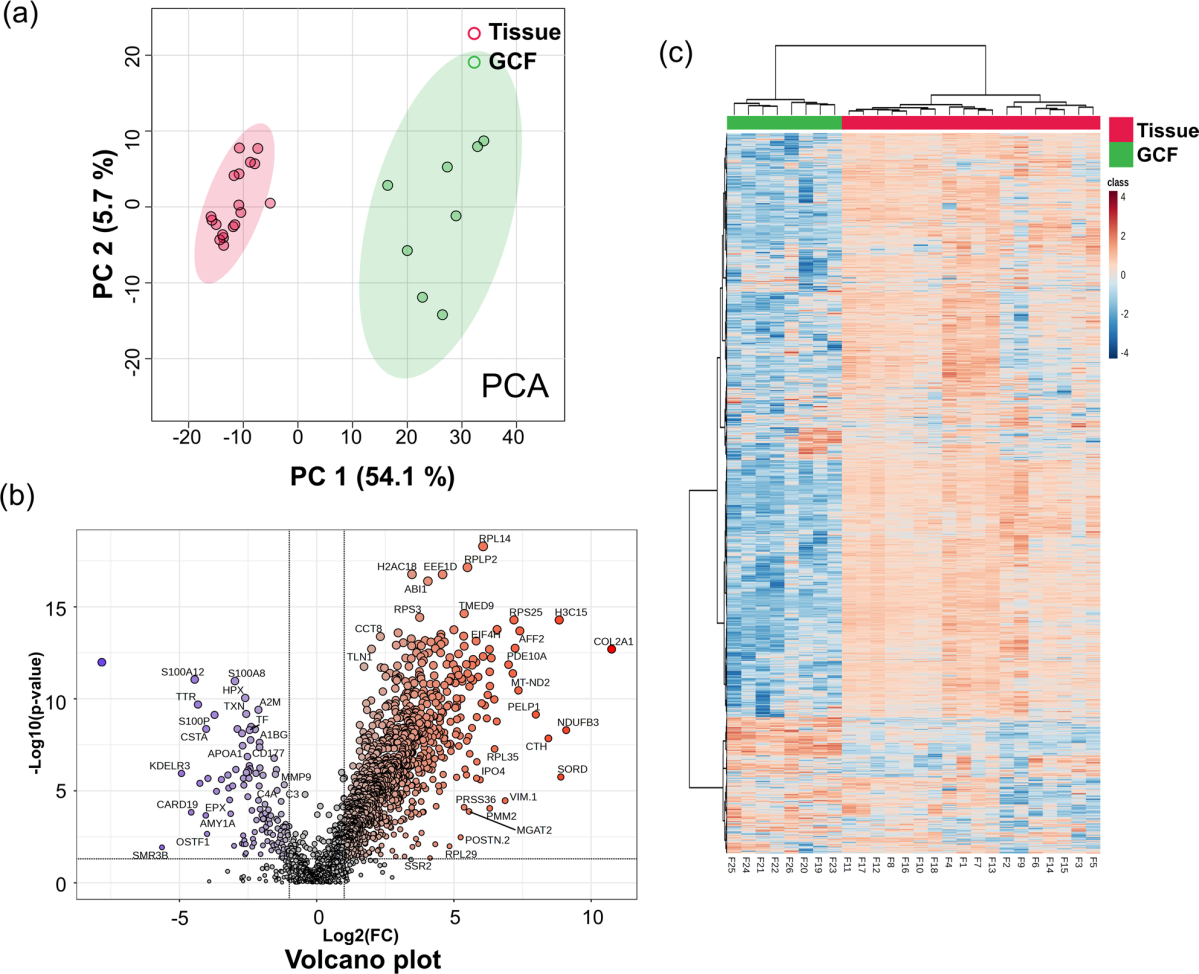
(**a**) Principal Component Analysis plot of tissue (red circle) and GCF (green circle) groups; PC 1 = 54.1%, component 2 = 4.8%. (**b**) Volcano plot of DEPs (Tissue/GCF). (**c**) Heatmaps representing the tissue and GCF group datasets. The color scale indicates the relative abundance of the proteins, with red color indicating increased levels and blue color indicating decreased levels

Furthermore, we conducted additional comparisons between the disease group to determine whether these changes were induced by the sample type or disease status. To minimize the differences caused by the control samples, we performed further differential expression analyses between the disease group samples. First, Principal Component Analysis (PCA) was performed to assess group differences. The PCA plot (Fig. 3a) showed PC 1: 54.1% and PC 2: 5.7%, indicating a distinct separation between the proteomes of periodontal tissue and GCF.

Our approach prioritized upregulated proteins to highlight molecular features potentially associated with ongoing inflammation, particularly in GCF, where exudative contents have been proposed to reflect local immune responses during disease progression [31]. For instance, previous studies have reported increased expression of inflammation-related proteins in GCF from patients with periodontitis [32, 33]. This approach aimed to emphasize proteins that are more reliably detectable and reflective of ongoing inflammatory activity within the sample type, which may also offer practical advantages for subsequent translational studies. DEPs were identified with |FC| > 2.0 (tissue/GCF) and p-value less than 0.05 (using Benjamini-Hochberg correction for FDR). A total of 1,244 DEPs were found among 1,117 upregulated proteins in the tissue group and 127 upregulated proteins in the GCF group. The volcano plot (Fig. 3b) and heatmap (Fig. 3c) illustrate these quantitative patterns, reaffirming increased upregulation in the tissue.

**Fig. 4:**
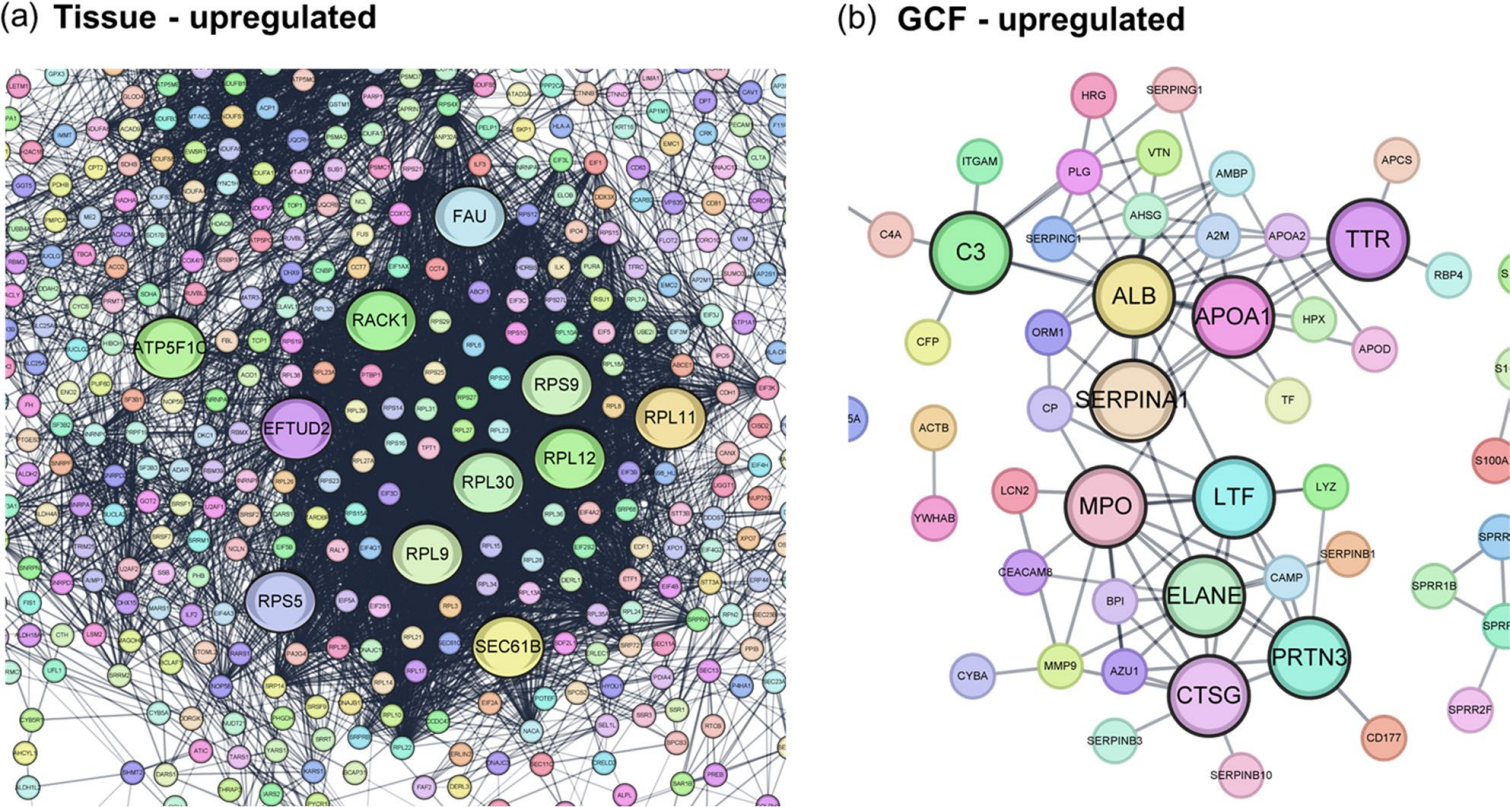
Each protein-protein interaction network was constructed based on the results of quantitative analysis using Cytoscape. Disconnected nodes were hidden to simplify the complex network view, and the network confidence was set to the highest scores (> 0.9). (**a**) Upregulated proteins in periodontal tissue. (**b**) Upregulated proteins in periodontal GCF. The top 10 hub-proteins in each network were selected using the CytoHubba plugin in Cytoscape to specify important nodes

To further identify the core regulatory proteins within each sample type, we conducted PPI network analysis of the DEPs, focusing on the upregulated proteins in each group. The PPI network of tissue-upregulated proteins identified 10 hub DEPs associated with cytoplasmic translation and ribosomal function: EEF1A1, RACK1, RPL11, RPL12, RPL23, RPL5, RPS11, RPS18, RPS27A, and RPS9 (Fig. 4a). The PPI network of GCF-upregulated proteins identified 11 hub DEPs: ALB, APOA1, APOE, C3, CTSG, ELANE, HP, LTF, MPO, SERPINA1, and TTR (Fig. 4b), which are involved in the defense response and serine-type endopeptidase activity.

### Identification of sample type-specific proteins

**Fig. 5:**
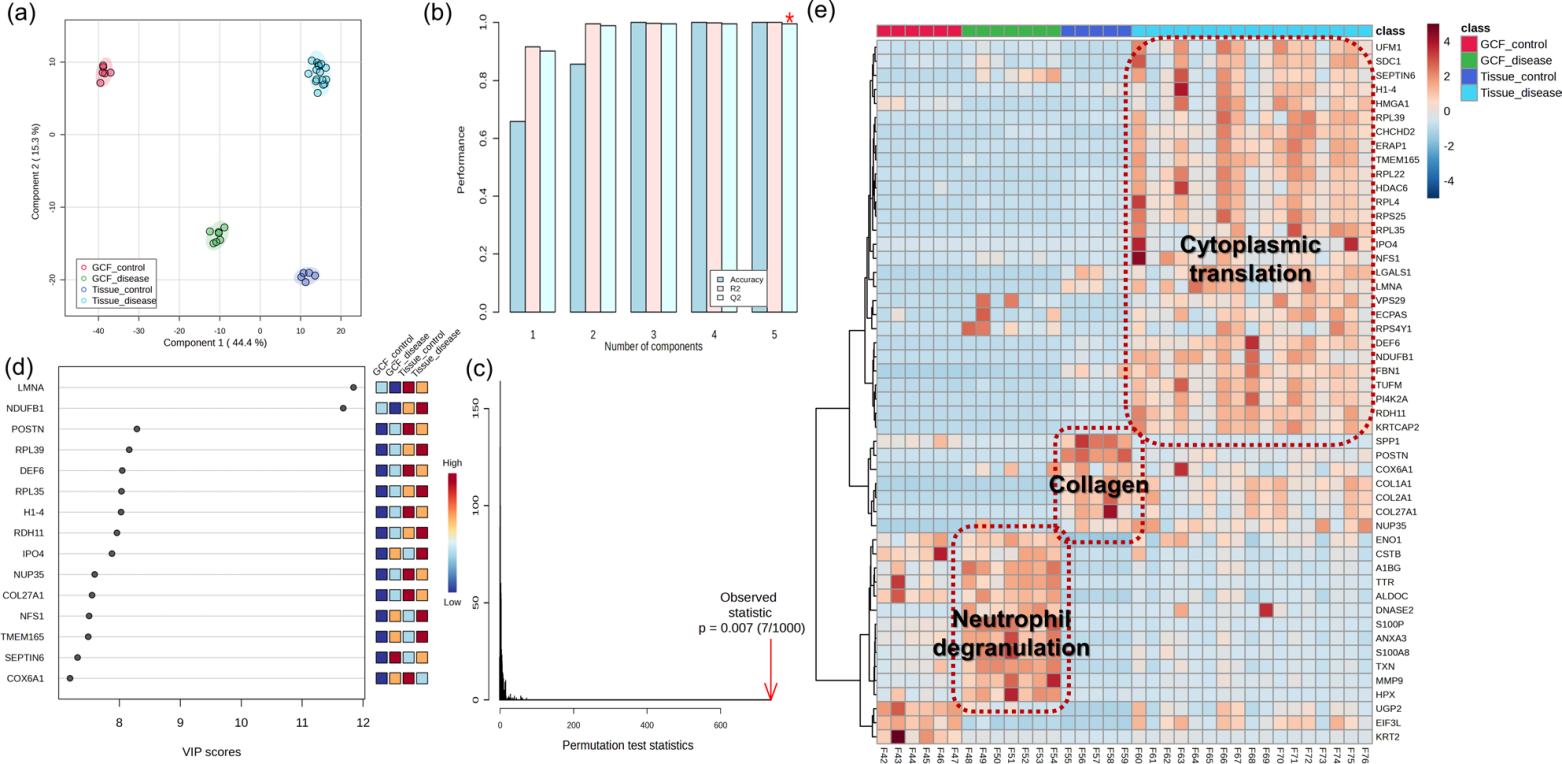
Comprehensive multivariate analysis and sample type-specific protein identification. (**a**) Partial Least Squares Discriminant Analysis plot showing distinct separation of four groups based on Component 1 (44.4%) and Component 2 (15.3%). (**b**) Model performance evaluation with Accuracy, R², and Q² values exceeding 0.95 at 5 components (asterisk). (**c**) Permutation test validation showing statistical significance (*p* = 0.007, 7/1000 permutations). (**d**) Variable Importance in Projection (VIP) scores for top discriminative proteins. LMNA, NDUFB1, POSTN, RPL39, and DEF6 show highest VIP scores (> 10). Adjacent bars indicate relative expression across groups. (**e**) Hierarchical clustering heatmap of top 50 proteins showing three functional clusters: Cytoplasmic translation (diseased periodontal tissue-specific), collagen (healthy tissue-specific), and neutrophil degranulation (diseased periodontal GCF-specific). Color scale represents high (red) to low (blue) expression

Our previous analyses independently confirmed sample type-specific molecular characteristics and differences between disease groups. However, comprehensive analysis incorporating all four groups was necessary to identify the proteins that most effectively distinguish GCF and tissue compartments among the numerous DEPs identified. Therefore, we performed PLS-DA and heatmap analysis encompassing all four groups to rank significant proteins and evaluate their contribution to molecular characterization. The PLS-DA plot showed that the four groups formed distinctly separated clusters based on Component 1 (44.4%) and Component 2 (15.3%) (Fig. 5a). Model performance evaluation showed high values above 0.95 for Accuracy, R², and Q² based on 5 components, and statistical significance was verified through permutation test (*p* = 0.007) (Fig. 5b, c). Variable importance in projection analysis identified LMNA, POSTN and others as top discriminative proteins, while RPL39 and NDUFB1 corresponded to previously identified ribosomal protein and NADH dehydrogenase pathways, respectively (Fig. 5d). Top 50 heatmap analysis reconfirmed the disease-specific expression of established biomarker S100A8 in periodontitis. In diseased periodontal tissue, ribosomal proteins (RPL39, RPL22, RPL4, RPL35, RPS25) and metabolic components (NDUFB1) demonstrated predominant upregulation, reinforcing their critical roles in tissue remodeling processes. In periodontitis GCF, neutrophil-associated proteins including S100P, MMP9, and ANXA3 showed specific upregulation, further confirming their importance as neutrophil-derived effectors in periodontal inflammation. These findings highlight the distinct pathophysiological characteristics of each sample type during disease progression (Fig. 5e).

## Discussion

In this study, we conducted a comprehensive proteomic analysis to characterize molecular signatures in periodontal disease and delineate the distinct roles of tissue and GCF compartments. Previous research has predominantly focused on individual sample types, such as GCF or salivary, limiting the comprehensive understanding of periodontitis and the development of integrated diagnostic strategies [34–36]. We examined disease-specific changes in each sample type relative to controls, then performed comparisons between tissue and GCF proteomes from periodontitis patients to delineate sample-type-specific characteristics. Analysis revealed coordinated upregulation of structural and metabolic pathways in periodontitis tissue, while GCF exhibited enhanced neutrophil-derived immune responses and bacterial defense mechanisms relative to controls. Comparative analysis between sample types further demonstrated that tissue and GCF represent functionally distinct compartments, with tissue enriched in structural remodeling proteins and GCF exhibiting immune signatures. This approach distinguished disease effects from sample-type differences, suggesting that GCF functions as an immune-enriched compartment rather than simple passive inflammatory accumulation. These findings provide molecular insights into both disease progression and distinct functional roles of periodontal compartments, reinforcing the potential of GCF as a non-invasive biomarker source.

### Disease-specific proteomic profiles

Analysis of disease-specific changes revealed that proteins involved in protein synthesis and cellular structure were predominantly upregulated in periodontal tissue. The identification of pathways related to collagen fibril and chromatin organizations, along with hub proteins including RPL11, COL6A1, and NDUFS1 from the five most notable WGCNA modules, indicates coordinated upregulation of structural and metabolic processes [37–41]. These findings encompassed ribosomal proteins involved in protein synthesis, collagen proteins for structural support, and NADH dehydrogenase proteins for energy production through oxidative phosphorylation [42–46]. The upregulation of translation elongation factor like EEF1A1 indicates enhanced protein synthesis, while mitochondrial components (NDUFS1 and IDH2) and extracellular matrix proteins such as COL6A1 and ASPN reflect increased metabolism and matrix remodeling processes. These findings demonstrate that despite the known activation of immune-related proteins in periodontal disease, abundance-based proteomic analysis by label-free quantitation shows that structural and metabolic protein networks appear predominantly in periodontal tissue compared to healthy controls. This molecular profile provides evidence that tissue-derived proteomics may be particularly suited for investigating structural therapeutic mechanism, wound healing pathways, and related biomarker targets rather than immune-focused biomarker discovery. These characteristics contrast with the immune-targeted protein profiles typically obtained through non-invasive sampling methods, indicating differentiated molecular contributions of tissue-based proteomic analysis in periodontal disease research [47].

In contrast to this repair-oriented tissue response, GCF exhibited distinct molecular characteristics centered on immune activation processes. The identification of defense-related modules M1 and M5 in GCF proteome revealed enrichment of antimicrobial effectors including S100A12 [48] and FCER1G [49], along with hub proteins such as S100A8 [50], and LCN2 [51] as neutrophil-derived proteins. These findings suggest that GCF captures regulated immune responses as its primary molecular signature, in marked contrast to proteome pattern observed in periodontal tissue. Additionally, metabolic modules M2 and M4 revealed downregulation of both glycolytic enzymes (PGK1, GAPDH) and proteins involved in translation and folding such as EEF1A1 and HSP90AB1, suggesting that GCF showed lower abundance of proteins associated with cellular metabolic processes.

To further investigate these distinct biological environments, network integration analysis of hub proteins revealed that GCF and tissue compartments might be connected primarily through housekeeping proteins (GAPDH, ALB, HSP90AB1), suggesting limited disease-specific molecular crosstalk. This network topology suggests that the immunologically active GCF environment and the repair-focused tissue compartment maintain largely distinct functional profiles despite their anatomical proximity. These findings support the rationale for developing environment-specific biomarker strategies rather than seeking universal periodontal markers, as the molecular signatures appear to reflect distinct aspects of periodontal pathophysiology, such as acute immune surveillance in GCF versus tissue remodeling in periodontal tissue. This finding emphasizes that GCF predominantly comprises an immunologically active biological environment. Moreover, these compartment-resolved molecular profiles may facilitate the translation of proteomic findings into clinically applicable biomarkers, providing a conceptual framework for precision periodontal diagnostics within translational proteomics through further validation studies.

### Sample-type-specific molecular characteristics

Comparative analysis between tissue and GCF from periodontitis patients supports compartment-specific molecular signatures that distinguish their respective biological roles in disease pathophysiology. Analysis focused on proteins significantly upregulated in each compartment. Upregulated proteins likely reflect active biological pathways, providing deeper insights into pathophysiology mechanisms [52, 53]. Additionally, this strategy is advantageous for biomarker exploration, as these proteins often demonstrate enhanced detectability and represent active disease processes. Comparative analysis between sample types revealed that neutrophil-derived effector proteins including MPO, ELANE, and CTSG, along with S100 family members and apolipoproteins, demonstrated significant enrichment in GCF, reflecting active inflammatory and immune regulatory mechanisms. Conversely, ribosomal proteins associated with protein synthesis machinery, alongside collagen and mitochondrial components, showed predominant upregulation in periodontal tissue, indicating structural remodeling and metabolic processes. This comparative analysis also revealed several DEPs beyond those identified in individual comparisons with controls, further indicating the potential presence of complexes contributing to mechanisms predominant in GCF such as S100 protein family and apolipoprotein networks. These findings underscore the need to develop biomarker discovery strategies that leverage molecular differences across sample types.

The neutrophil-derived proteomic profile of GCF revealed differential detection patterns, wherein MPO and LTF emerged exclusively from individual disease group analyses, while ELANE, CTSG, HP, and SERPINA1 demonstrated consistent identification across individual and comparative analyses [12, 54, 55]. These proteins, functioning in antimicrobial and acute phase responses and identified as hub proteins in PPI analysis, suggest coordinated expression patterns. This differential detection pattern indicates that these significant proteins may represent compartment-specific regulatory proteins that distinguish GCF from tissue. Given the established associations between periodontal disease and systemic inflammation, our findings suggest that GCF, as a plasma-derived exudate, may reflect broader inflammatory networks beyond simple accumulation of local tissue-derived responses [56]. These implications emphasize the necessity for strategies that consider molecular distinctions between periodontal tissue and GCF in biomarker exploration, although confirmation of extra-tissue origin requires further study.

These compartment-specific findings are further supported by the identification of S100 family proteins and apolipoproteins in GCF, with additional apolipoproteins such as APOA1 and APOD detected through comparative analysis. While some studies have reported associations between specific S100 proteins and apolipoproteins, or co-upregulation in GCF from periodontal patients, periodontal studies have rarely examined both protein groups within a single framework [57, 58]. This expression pattern provides additional evidence for our characterization of GCF as an immune-focused biological environment, contrasting with the structural and metabolic protein signatures predominant in periodontal tissue. While the precise mechanisms underlying these interactions require independent validation studies, the identification of these protein networks through our comparative approach demonstrates molecular distinctions between sample types.

Finally, our four-group comparative analysis identified compartment-specific biomarker candidates for each periodontal sample type. The integrated analytical approach validated established molecular signatures and identified additional disease-specific proteins. Notably, the confirmation of S100A8 expression patterns across all analytical frameworks supports its potential as a GCF-based marker, while the identification of ribosomal protein networks (RPL39, RPL22, RPL4, RPL35, RPS25) and metabolic components (NDUFB1) in diseased tissue provides molecular basis for tissue-specific approaches. Furthermore, the identification of neutrophil-associated proteins including S100P, MMP9, and ANXA3 in periodontitis GCF adds to the characterized immune effector repertoire. These findings establish distinct molecular profiles for periodontal tissue and GCF compartments, providing sample type-specific biomarker candidates for targeted diagnostic approaches.

However, several study limitations should be acknowledged with corresponding mitigation strategies. Sample sizes were limited, reducing statistical power. We therefore focused on DEPs with stringent statistical criteria, though smaller-effect proteins may have been missed. Future studies with larger cohorts are needed for comprehensive proteomic profiling. Next, periodontal tissue and GCF samples were collected from distinct patient cohorts. Thus, we recruited both cohorts using strict exclusion criteria and clinical parameters to ensure periodontitis with comparable disease severity. Also, heat stabilization process applied to the tissue samples was not used for GCF, several differences may affect in protein detection, particularly for enzymes. To mitigate this, standardized extraction and LC-MS protocols were applied consistently across all samples to minimize technical variation. Finally, the cross-sectional design restricts causal inference, though the robust statistical framework including WGCNA and PPI network analysis supported the consistency of observed proteomic associations.

## Conclusion

This comparative proteomic analysis reveals that periodontal tissue and GCF represent functionally distinct compartments with sample type-specific molecular characteristics in periodontal disease. Periodontal tissue demonstrated coordinated upregulation of structural and metabolic processes including ribosomal proteins and NADH dehydrogenase components, while GCF exhibited enrichment of neutrophil-derived proteins and antimicrobial effectors as its primary molecular signature. Integration analysis revealed that these compartments maintain largely distinct functional profiles despite their anatomical proximity, suggesting that GCF comprises an immunologically active biological environment rather than simple passive inflammatory accumulation. These molecular distinctions provide evidence for developing compartment-specific diagnostic strategies that bridge molecular pathology and clinical diagnosis, highlighting the translational relevance of this comparative proteomic approach in periodontal disease.

## Supplementary Information

Supplementary Material 1

Supplementary Material 2

Supplementary Material 3

## Data Availability

The mass spectrometry proteomic data that support the findings of this study are publicly available at the ProteomeXchange Consortium (https://proteomecentral.proteomexchange.org/) via the PRIDE partner repository with the dataset identifier PXD054953 (project webpage: https://proteomecentral.proteomexchange.org/cgi/GetDataset? ID=PXD054953) [59]. Reviewer account details: Username: reviewer\_pxd054953@ebi.ac.uk; password: mPLPAsGQPBwX.

## Footnotes

1 ABCAmmonium Bicarbonate. BHBenjamini‑Hochberg correction. BOPBleeding on Probing. DAVIDDatabase for Annotation, Visualization and Integrated Discovery. DEP(s)Differentially Expressed Protein(s). FAFormic acid. FCFold Change. GCFGingival Crevicular Fluid. IAAIodoacetamide. kMEModule Eigengene‑based Connectivity. MCODEMolecular Complex Detection. PDProbing Depth. PPIProtein–Protein Interaction. STRINGSearch Tool for the Retrieval of Interacting Genes/Proteins. TCEPTris(2‑carboxyethyl)phosphine. UniProtUniversal Protein Resource. WGCNAWeighted Gene Co‑expression Network Analysis.
