# Supplementary figures and images for: Comparative proteomic analysis of gingival crevicular fluid and periodontal tissue: revealing clinical potential
Source: Clin Proteomics. 2026 Feb 24;23:16. doi: 10.1186/s12014-026-09587-3 (PMC13037294; doi:10.1186/s12014-026-09587-3)

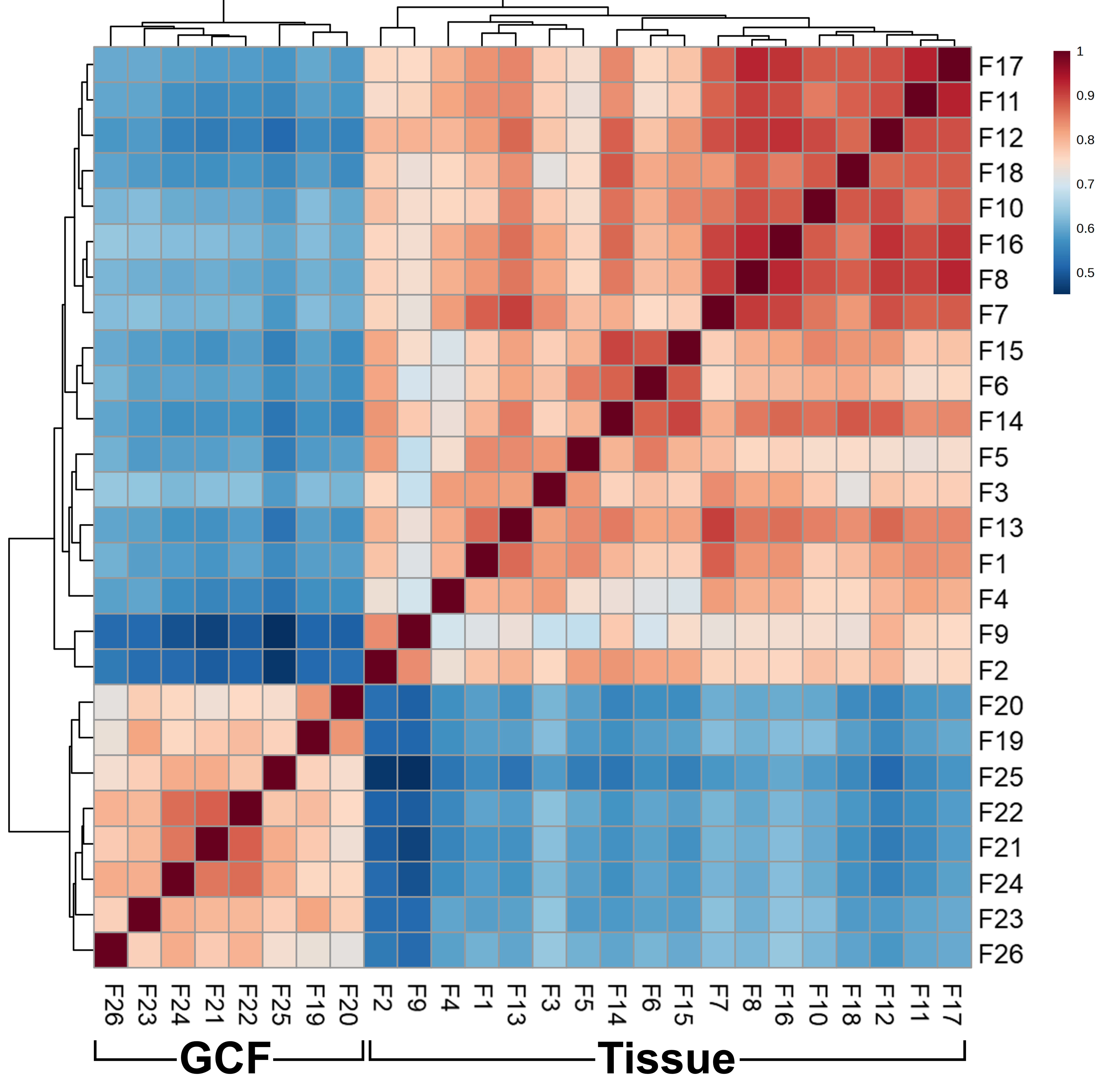

Supplement: Supplementary file 3 — Supplementary Material 3 [file 12014_2026_9587_MOESM3_ESM.tif]
